# Preoperative Diagnosis of Periprosthetic Infection in Patients Undergoing Hip or Knee Revision Arthroplasties: Development and Validation of Machine Learning Algorithm

**DOI:** 10.3390/diagnostics15050539

**Published:** 2025-02-23

**Authors:** Vincenzo Di Matteo, Pierandrea Morandini, Victor Savevski, Guido Grappiolo, Mattia Loppini

**Affiliations:** 1 Department of Biomedical Sciences, Humanitas University, Pieve Emanuele, 20090 Milan, Italy; vincenzo.dimatteo@st.hunimed.eu; 2 IRCCS Humanitas Research Hospital, Rozzano, 20089 Milan, Italy; guido.grappiolo@me.com; 3 Artificial Intelligence Center, IRCCS Humanitas Research Hospital, Via Manzoni 56, Rozzano, 20089 Milan, Italy; pierandrea.morandini@humanitas.it (P.M.); victor.savevski@humanitas.it (V.S.)

**Keywords:** artificial intelligence, arthroplasty, deep learning, machine learning, hip, knee, periprosthetic joint infection

## Abstract

**Background:** Periprosthetic joint infection (PJI) remains a significant and complex complication following total hip and knee arthroplasty. This study aims to design, validate, and assess a machine learning (ML) model for predicting the likelihood of PJI in individuals undergoing revision arthroplasty procedures. **Methods:** A retrospective analysis was conducted on patients who underwent hip or knee revision arthroplasty between 1 January 2015 and 31 March 2021. Data were collected from preoperative clinical histories, laboratory results, and patient demographics. The final dataset was used to train multiple classification models for the preoperative prediction of PJI. **Results:** A total of 1360 patients were included, comprising 1141 cases in the aseptic group and 219 in the infected group. The best-performing model, a Linear Support Vector Machine (SVM), demonstrated reasonable predictive capability for PJI, achieving an area under the curve (AUC) of 0.770 ± 0.008 in the training set and 0.730 ± 0.078 in the testing set. Additionally, three key predictors of PJI were identified. **Conclusions:** The Linear SVM model, developed using preoperative clinical information, exhibited reasonable performance in predicting PJI. While further refinement and validation are necessary, integrating ML tools into the preoperative evaluation process has the potential to enhance personalized risk assessment, support informed decision-making, and optimize surgical preparation for patients undergoing prosthetic revision surgery.

## 1. Introduction

Periprosthetic joint infection (PJI) accounts for 15% of failed total hip arthroplasties (THAs) and for 25% of failed total knee arthroplasties (TKAs) [1]. PJI occurs after 2% of primary THA or TKA [2], which translates to around 3000 cases annually in Italy [3]. This number is expected to rise due to the increasing number of primary procedures performed [2,3]. Preoperative PJI diagnosis remains a clinical challenge, as there is no universally accepted gold standard, particularly in cases caused by low-virulence organisms or in immunocompromised individuals. Currently, no single system effectively ensures preoperative PJI diagnosis. In clinical practice, both preoperative and intraoperative investigations are commonly used [4]. Several diagnostic criteria have been developed, including those proposed by the Musculoskeletal Infection Society (MSIS), the International Consensus Meeting (ICM), the Infectious Disease Society of America (IDSA), and the European Bone and Joint Infection Society (EBJIS) [4]. The diagnosis of periprosthetic joint infection (PJI) typically requires postoperative confirmation through the isolation of a pathogen from intraoperative cultures. However, in some cases, PJI is diagnosed incidentally through routine microbiological tests performed during revision procedures, even in the absence of clinical suspicion. As a result, the true incidence of PJI is likely higher than what is reported in official registries. Additionally, bacterial biofilm formation can hinder the detection of infection during microbiological analysis, leading to a 15% false-negative rate due to impaired test sensitivity [5]. Various microbiological detection techniques have been developed, such as sonication, synovial sampling, and incubation period prolongation [6,7]. While these advancements have enhanced microbiological sensitivity, they remain too costly for routine clinical use. This underscores the need for a system that can diagnose PJI preoperatively without reliance on intraoperative investigations or postoperative confirmation. In recent years, artificial intelligence (AI) has made significant strides, particularly through the application of powerful computing and deep learning algorithms for predicting surgical outcomes. Machine learning (ML), a subset of AI, represents a natural evolution of traditional statistical modeling [8,9]. This scientific discipline focuses on how computers learn from data, imitate human thinking, and may overcome human capabilities [10]. ML is becoming a promising and powerful technology in the prevention of PJI, involving developing and using algorithms capable of identifying correlations and patterns of complex risk factors; it may benefit the prediction, diagnosis, treatment, and prognosis of PJI [11]. While some ML models have been explored for PJI prevention, relatively few studies have investigated their application in this domain. To the best of our knowledge, this is the first study to use ML models for the preoperative prediction of PJI in patients undergoing revision total hip arthroplasty (r-THA) and revision total knee arthroplasty (r-TKA) at a high-volume, single-center institution. The purpose of this retrospective observational cohort study is to develop, validate, and evaluate an ML-based prediction tool that utilizes preoperative patient-specific clinical objective criteria. The multivariable Linear Support Vector Machine (SVM) model was used to predict preoperative PJI in patients undergoing r-THA and r-TKA in a high-volume center.

## 2. Materials and Methods

This study was conducted in accordance with the Declaration of Helsinki and good clinical practice guidelines. The study protocol for the development of this registry was approved by the Ethics Committee of Humanitas Research Hospital IRCCS (protocol code 444/21) in May 2021. Given the retrospective nature of the study, all included patients had previously provided written informed consent to be included in the registry of orthopedic surgical procedures, granting permission for the use of their clinical data for research purposes.

### 2.1. Data Extraction

The algorithm was developed using clinical data extracted from the electronic health records of patients who underwent r-THA and r-TKA between 1 January 2015 and 31 March 2021 in our Orthopedics Department. Data extraction was performed by querying the Humanitas Data Warehouse (DWH) to retrieve relevant patient information. The available dataset included vital parameters and laboratory test results collected during pre-admission assessments.

### 2.2. Data Selection and Inclusion and Exclusion Criteria

This retrospective observational cohort study included patients who underwent elective r-THA or r-TKA performed by senior surgeons experienced in joint replacement surgery between 1 January 2015 and 31 March 2021. Patients were identified from hospital clinical records using the International Classification of Diseases, Ninth Revision, Clinical Modification (ICD-9-CM) (procedure codes: 00.70, 00.71, 00.72, 00.73 for revision THA; 80.06, 81.55, 00.80, 00.81, 00.82, 00.83, 00.84 for revision TKA). Eligibility criteria included patients undergoing elective revision THA or TKA in our Orthopaedic Department with all the required predictive features recorded. The exclusion criteria were patients undergoing elective primary THA or TKA (International Classification of Diseases, Ninth Revision, Clinical Modification (ICD9-CM); procedure codes: 81.51 for primary THA and 81.54 for primary TKA), patients aged under 18 years old, and patients who did not have all the required predictive features recorded. All included patients were further assessed using ICD-9-CM diagnostic codes (99666 and 99667) for infection and inflammatory reactions related to joint prostheses, along with postoperative culture-positive sampling for prosthetic joint infections. Based on these criteria, the cohort was divided into an aseptic group and a septic group.

### 2.3. Feature Selection

As a first step, all variables with a quote of missing values above or equal to 25% were removed. Then, a feature selection phase was performed, aiming to reduce the number of input features for the classification algorithm. To identify the most relevant features for the analysis, a Random Forest ensemble classifier was employed as the feature selector. This method was preferred among all others since it is more robust to noise or irrelevant features due to its ensemble nature. Also, the non-linear behavior of Random Forest allows it to select more insightful and non-redundant predictors compared to more traditional *p*-value-based univariate analysis. Additionally, Random Forests naturally account for feature interactions through their hierarchical splitting process, providing a more comprehensive assessment of feature importance in the context of other variables [12]. The model was configured with a maximum depth of 3 nodes and up to 15 leaves per node, ensuring a balance between model complexity and interpretability. This setup was chosen to focus on capturing meaningful interactions between features without overfitting. Feature importance scores were derived from the trained Random Forest model, ranking the contribution of each feature to the predictive task. From this ranking, the top 20 features with the highest importance scores were selected for further analysis, ensuring that only the most informative variables were included in subsequent modeling. 

### 2.4. Classification

The study evaluated multiple classification models to determine the most suitable approach for the analysis. The candidate models, all implemented using the Python sklearn library version 1.2.2 [13], included Logistic Regression, Gradient-Boosted Classification Trees, Random Forest, Linear SVM, Gaussian SVM, and K-Nearest Neighbor.

The classification pipeline consisted of four sequential stages. First, missing values were addressed through mean imputation based on the overall population. Second, features were normalized using min–max scaling. Third, a feature selection process identified and retained the most informative variables. Finally, the selected classification model was applied to the processed data.

Model optimization was performed using a randomized hyperparameter search, with logistic loss serving as the optimization metric. The final model’s performance was evaluated using 10-fold cross-validation.

### 2.5. Statistical Analysis

The statistical analysis primarily focused on extracting descriptive statistics of the available features. *t*-tests were applied to numerical features, while Chi-Squared tests were used for binary features.

To further assess the relevance of the selected features, an odds ratio (OR) analysis was conducted. This analysis focused on features that were selected in at least 7 out of the 10 folds during cross-validation, ensuring that only consistently important features were evaluated. For each feature, the odds ratio and corresponding 95% confidence intervals were calculated to quantify the strength and direction of the association between the feature and the outcome variable. Continuous features are described as “median [Q1 Q3]”, and binary variables are described as “absolute-count (percentage)”. 

## 3. Results

### 3.1. Dataset

Overall, 1360 patients were enrolled (57.7% women, median age 66.6 [56.9 Q1–75.1 Q3]). Of these, 1141 patients in the aseptic cohort (60.6% women, median age 66.6 [56.8 Q1–75.1 Q3]) and 219 patients in the septic cohort (42.9% women, median age 67.1 [57.7 Q1–75.0 Q3]) were, respectively, included. The second surgeries were mainly performed on the hips: 1231 (90.5%) in total, 1047 (91.8%) in the aseptic cohort, and 184 (84.0%) in the septic cohort. The selected features are mostly laboratory exams and demographic features. Table 1 shows the feature distribution in the extracted dataset [14]. 

### 3.2. Classification

The classification analysis addressed an imbalanced dataset (1141 aseptic patients versus 219 infected patients) by implementing balanced class weights to increase the penalty for misclassification of infected cases. Model performance was evaluated using precision, recall, and F1 score metrics through 10-fold cross-validation, with the results aggregated using a macro-averaging approach. Among all the tested models, Linear SVM demonstrated superior performance (Table 2). Linear models outperformed ensemble classifiers, likely due to their more robust representation capabilities. In contrast, ensemble models showed signs of overfitting on the training data, resulting in decreased test set performance.

Detailed analysis of the Linear SVM performance (Table 3) revealed consistent metrics across both the training and test sets for both cohorts. The aseptic cohort achieved F1 scores of 0.841 ± 0.034 and 0.826 ± 0.093 in the training and test sets, respectively, while the infected cohort showed F1 scores of 0.462 ± 0.021 and 0.446 ± 0.074. This consistency indicates successful model generalization and minimal overfitting risk.

**Figure 1:**
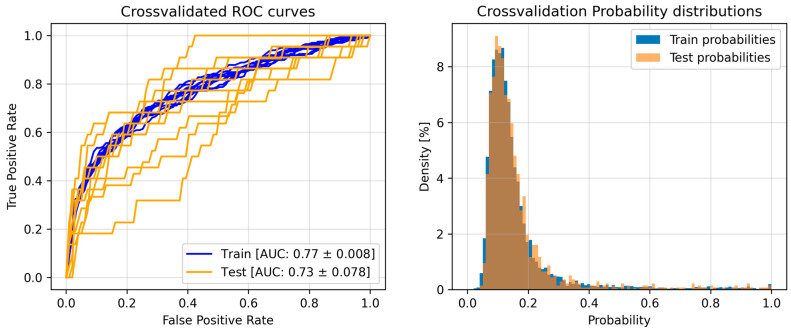
ROC curves and the distribution of the predicted probabilities.

The model’s discriminative ability is illustrated in Figure 1 through ROC curves and probability distributions. The AUC values (0.77 ± 0.008 for training and 0.73 ± 0.078 for testing) and similar probability distributions between sets further support the model’s generalization capabilities.

**Figure 2:**
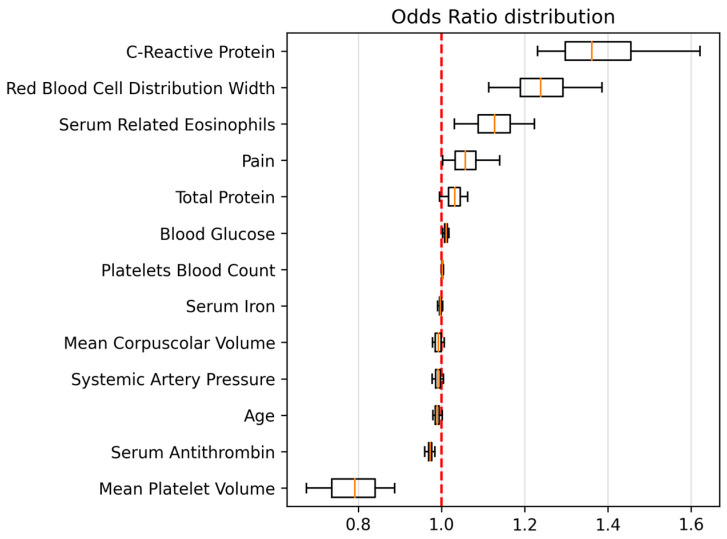
Multivariate logistic regression odds ratios and 95% CIs.

Feature importance was assessed through odds ratio analysis using an unregularized Logistic Regression model (Figure 2). This analysis focused on features selected in at least 7 of the 10 cross-validation folds. The results highlighted serum C-reactive protein (CRP), a systemic inflammation marker, as the strongest positive predictor of periprosthetic joint infection (PJI).

## 4. Discussion

Artificial intelligence (AI) has demonstrated great potential in enhancing diagnostic accuracy and efficiency while also aiding in the identification of risk factors for various diseases. This study evaluated the preoperative use of machine learning (ML) algorithms to predict periprosthetic joint infection (PJI) in patients undergoing revision total hip arthroplasty (r-THA) and revision total knee arthroplasty (r-TKA) using available preoperative clinical data. The most notable finding of this study is that the ML algorithm demonstrated good discriminatory performance in predicting PJI among the selected patients, with an area under the curve (AUC) of 0.770 ± 0.008 in the training set and 0.730 ± 0.078 in the test set. The strongest predictors of PJI were systemic markers of inflammation, including serum C-reactive protein (CRP), serum red blood cell distribution width (RDW) and serum-related eosinophils. These readily available preoperative markers showed a positive correlation with PJI occurrence. Notably, the erythrocyte sedimentation rate (ESR), a validated systemic inflammation marker, was not included as a feature due to it having over 25% missing values. ML models may be improved by incorporating clinically relevant variables, regarding PJI as a joint local phenomenon rather than a systemic process. Such variables include synovial white blood cell (WBC) count and synovial polymorphonuclear neutrophils (PMNs), which can be obtained through preoperative joint aspiration and synovial fluid analysis. Currently, surgeons and internal medicine physicians seeking to diagnose PJI use a multidisciplinary test battery that includes tests to detect joint local inflammation, such as synovial fluid white blood cell (WBC) count and synovial tissue histology [15]. The analysis of synovial fluid obtained by preoperative joint aspiration, including total cell count, differential leucocyte count, and cultures for aerobic and anaerobic organisms, has shown sufficient sensitivity and specificity in multiple studies. It is the most valuable diagnostic tool and should be performed prior to each surgical revision procedure [4]. In our hospital, no preoperative synovial fluid analysis was performed; therefore, these interesting features were not reported. Regarding features related to the formation of cutaneous fistulae and/or the drainage of purulent secretions, the present study investigated features related to misdiagnosed “silent” PJI; hence, fistulae and purulent drainage were not considered. Moreover, a previous study reported that the rate of developing a sinus tract in PJI was 21.3%. The presence of a sinus tract may be a proxy for other issues, such as poor periarticular soft tissue, poor nutritional status of the host, and multiple prior operations [16]. A PJI complication is estimated to occur in 1–3% of patients undergoing primary replacement and in 3–5% of patients undergoing revision [17]. With 219 patients in the septic cohort and 1141 patients in the aseptic cohort, the patient sample was representative and aligned with the existing literature [1]. With the increasing adoption of joint replacement surgery and advancements in related technologies, a growing number of patients are undergoing these procedures. Currently, there is no universal guideline for PJI prediction after r-THA or r-TKA. In clinical practice, patients at high risk of developing PJI after r-THA or r-TKA are identified based on the presence or absence of risk factors, and clinicians can only predict the risk of PJI based on their experience. When a patient’s condition is complex or the dataset is incomplete, the difficulty of prediction may increase, requiring more time for an accurate assessment.

The machine learning algorithm presented in this study may be a promising alternative to the manual risk prediction method in preoperatively predicting PJI in patients undergoing r-THA or r-TKA. The tool presented here can forecast outcomes for patients with PJI before surgery; the strongest predictors of PJI occurrence were high levels of serum CRP, serum RDW, and serum-related eosinophils. In recent years, medical researchers have increasingly employed ML applications in the prevention of PJI. The models were divided into four categories: prediction, diagnosis, antibiotic application, and prognosis.

Yeo et al., in 2022, investigated the use of Artificial Neural Networks for predicting superficial surgical site infections and PJI following TKA. They retrospectively included 10,021 consecutive primary TKA patients; the average follow-up time lasted about 2.8 years. SSIs were reported in 404 (4.0%) TKA patients, including 223 superficial surgical site infections and 181 PJIs. The patients’ demographic and operational variables were collected. The model performance was good, with an AUC of 0.84 and a Brier score of 0.054 (a Brier score close to zero indicates good accuracy of probabilistic prediction). The strongest predictors of surgical site infections following primary TKA were the Charlson comorbidity index, obesity (BMI >30 kg/m^2^), and smoking. The neural network model represented an accurate method to predict patient-specific superficial surgical site infections and PJI following primary TKA [18]. Klemt et al., in 2021, used artificial intelligence to evaluate prognostic outcomes. They retrospectively reviewed 618 r-TKA procedures for PJI. They demonstrated an ML model with excellent performance for predicting recurrent infections in patients following r-TKA for PJI. The ML models achieved excellent performance across discrimination (AUC range: 0.81–0.84), with a Brier score of 0.053. The strongest predictors for recurrent PJI in patients following r-TKA included irrigation and debridement with or without modular component exchange (*p* < 0.001), > 4 prior open surgeries (*p* < 0.001), metastatic disease (*p* < 0.001), drug abuse (*p* < 0.001), HIV/AIDS (*p* < 0.01), the presence of Enterococcus species (*p* < 0.01), and obesity (*p* < 0.01) [19]. Tao et al., in 2022, trained a deep learning model to diagnose PJI. R-TKA patients were enrolled from Chinese People’s Liberation Army General Hospital. PJI was based on the 2018 ICM guidelines. Frozen pathological sections were converted into electronic images, with 461 positive and 461 negative images used for model training. The model achieved an AUC of 0.814 and an average accuracy of 93.3% [20]. Wu et al. developed an accurate machine learning model using administrative and electronic medical records (EMRs) to improve surgical site infection (SSI) detection accuracy. The study cohort consisted of 16,561 primary TKA and 10,799 primary THA cases retrospectively included. Their findings suggested that ML models derived from administrative data and EMR text data achieved high performance and could be used to automate complex SSI detection, with an ROC AUC of 0.906 (95% CI 0.835–0.978), PR AUC of 0.637 (95% CI 0.528–0.746), and F1 score of 0.790 (0.670–0.900) [21]. Sapienza et al., in 2024, reported the importance of considering patient-specific factors such as bone quality and comorbidities when selecting the type of prosthetic implant [22], and they also stated that a machine learning model could be very useful in guiding the orthopedic surgeon in choosing the most suitable prosthetic implant during prosthetic revision for PJI. Zhu et al., in 2025, reported the efficacy of an artificial intelligence preoperative planning system for assisting in revision surgery; the results of this study showed that artificial intelligence preoperative planning had high accuracy in predicting prosthesis models in hip revision surgery, increasing the accuracy of preoperative planning, reducing the intraoperative error rate, and improving surgical efficiency [23]. 

This study’s findings should be interpreted in light of its limitations. First, as a retrospective study, it is subject to inherent biases and limitations in data reporting. Second, it was conducted at a single institution, which may introduce confounding effects due to unmeasured variables and limit its generalizability to other clinical settings. Third, while the minimum follow-up period was two years, a longer follow-up may capture additional recurrent infections and re-revisions. Lastly, the sample size, particularly for infected cases, is relatively small, leading to a pronounced class imbalance that posed challenges for model training.

Despite these limitations, this study provides valuable insights into the feasibility of machine learning for PJI prediction. The centralized patient selection from a high-volume prosthetic surgery center enhances the homogeneity of perioperative treatment, ensuring a more controlled and specific analysis. The model demonstrated reasonable discriminatory ability (AUC = 0.73) despite the complexity of PJI diagnosis and the challenges posed by imbalanced data. While the current model may not yet be ready for clinical implementation, it represents an important step toward developing machine learning-based decision support tools. Future studies should focus on improving predictive performance by incorporating additional clinical and biomarker features, utilizing larger multi-institutional datasets, and exploring alternative modeling approaches. Prospective multicenter studies with long-term follow-up are needed to refine and validate these models, ultimately enhancing their accuracy and clinical applicability in PJI prediction.

## 5. Conclusions

This study developed and validated a machine learning model to preoperatively predict PJI in patients undergoing r-THA or r-TKA. The results demonstrate that linear models, particularly SVM and Logistic Regression, achieved reasonable performance despite the challenges posed by class imbalance and the complexity of PJI diagnosis. While the model is not yet ready for clinical implementation, it highlights the potential of artificial intelligence in leveraging preoperative clinical data to support PJI prediction. The results suggest that there is potential to integrate computerized algorithms into electronic health record systems, where they may be employed to assist in clinical decision-making, supplementing the ICM criteria for PJI prediction. Further, prospective studies are mandatory to implement our findings and validate the reliability of this technology in clinical practice.

## Figures and Tables

**Table 1:** Summary of patient features: in bold are highlighted the features selected more than 7 times during the cross-validation.

Features		Missing	Overall	0	1	*p*-Value
Number			1360	1141	219	
**Age**, mean (SD)		0	65.1 (13.1)	65.0 (13.3)	65.7 (12.0)	0.421
Gender, n (%)	Male		575 (42.3)	450 (39.4)	125 (57.1)	<0.001
	Female		785 (57.7)	691 (60.6)	94 (42.9)	
Prothesis type, n (%)	Knee		129 (9.5)	94 (8.2)	35 (16.0)	0.001
	Hip		1231 (90.5)	1047 (91.8)	184 (84.0)	
**Pain**, mean (SD)		1	0.8 (0.4)	0.8 (0.4)	0.9 (0.4)	0.017
Breath Rate, mean (SD)		69	15.8 (1.2)	15.7 (1.2)	15.9 (1.1)	0.154
Heart Rate, mean (SD)		1	74.4 (7.2)	74.4 (7.1)	74.6 (7.8)	0.725
**Systemic Artery Pressure**, mean (SD)		1	120.1 (10.2)	120.1 (10.2)	120.2 (10.1)	0.923
Oxygen Saturation Index, mean (SD)		1	98.0 (1.1)	98.0 (1.1)	97.9 (1.0)	0.251
Temperature, mean (SD)		1	36.4 (0.2)	36.4 (0.2)	36.4 (0.2)	0.009
**Serum Antithrombin**, mean (SD)		45	99.7 (12.7)	100.3 (12.8)	96.5 (11.7)	<0.001
**Serum Related Eosinophils**, mean (SD)		0	2.6 (1.8)	2.5 (1.7)	3.0 (2.2)	0.005
Serum Hematocrit, mean (SD)		0	41.7 (4.0)	41.9 (3.8)	40.5 (4.5)	<0.001
Serum-Related Lymphocytes, mean (SD)		0	28.5 (8.2)	28.9 (8.3)	26.0 (7.6)	<0.001
**Mean Corpuscolar Volume**, mean (SD)		0	88.7 (6.8)	88.9 (6.7)	87.3 (6.7)	0.001
**Mean Platelet Volume**, mean (SD)		0	8.9 (1.0)	8.9 (1.0)	8.5 (1.0)	<0.001
**Serum Iron**, mean (SD)		43	80.0 (31.5)	82.3 (30.4)	68.4 (34.1)	<0.001
**Blood Glucose**, mean (SD)		203	100.0 (18.8)	99.4 (17.9)	103.7 (23.2)	0.020
**C-Reactive Protein**, mean (SD)		114	0.7 (1.3)	0.6 (1.0)	1.5 (2.1)	<0.001
**Total Protein**, mean (SD)		0	11.9 (3.5)	11.8 (3.2)	12.9 (4.5)	0.001
Serum Sodium, mean (SD)		201	141.8 (2.2)	141.9 (2.2)	141.4 (2.0)	0.012
**Red Blood Cell Distribution Width**, mean (SD)		0	14.4 (1.4)	14.3 (1.3)	15.1 (1.9)	<0.001
**Platelet Blood Count**, mean (SD)		0	242.7 (68.4)	237.8 (62.3)	268.0 (90.3)	<0.001

**Table 2:** Performances comparison table across classification models. Metrics were computed for both train and test sets using 10-fold cross-validation, with macro-averaging applied across aseptic and infected classes.

	Test			Train		
	F1	Precision	Recall	F1	Precision	Recall
**Linear SVM**	0.636	0.644	0.692	0.652	0.643	0.712
**Logistic Regression**	0.632	0.639	0.684	0.65	0.639	0.712
**Gradient-Boosted Trees**	0.632	0.654	0.679	0.71	0.693	0.809
**Random Forest**	0.632	0.685	0.674	0.663	0.653	0.72
**Gaussian SVM**	0.628	0.634	0.688	0.729	0.706	0.774
**KNN**	0.596	0.618	0.615	0.703	0.689	0.723

**Table 3:** Model classification scores for the two classes.

	Train		Test	
	Aseptic	Septic	Aseptic	Septic
F1	0.841 ± 0.033	0.462 ± 0.021	0.826 ± 0.093	0.446 ± 0.074
Precision	0.921 ± 0.008	0.365 ± 0.046	0.914 ± 0.028	0.373 ± 0.096
Recall	0.775 ± 0.063	0.649 ± 0.069	0.767 ± 0.146	0.616 ± 0.157

## Data Availability

The original contributions presented in this study are included in the article. Further inquiries can be directed to the corresponding author.
